# Oral hygiene knowledge versus behavior in children: A questionnaire‐based, interview‐style analysis and on‐site assessment of toothbrushing practices

**DOI:** 10.1002/cre2.607

**Published:** 2022-06-17

**Authors:** Madline P. Gund, Marina Bucher, Matthias Hannig, Tilman R. Rohrer, Stefan Rupf

**Affiliations:** ^1^ Clinic Department of Operative Dentistry, Periodontology and Preventive Dentistry Saarland University Homburg Germany; ^2^ University Children's Hospital, Saarland University Medical Centre Homburg Germany; ^3^ Department of Synoptic Dentistry Saarland University Homburg Germany

**Keywords:** behavior, dental hygiene, knowledge, questionnaire

## Abstract

**Objectives:**

Oral hygiene plays an important role in eliminating biofilms and preventing dental caries. However, the implementation of oral health knowledge that children learn from their parents and through school dental health programmes remains poorly studied. This study aimed to investigate oral hygiene knowledge and its practical utilization in children and young adolescents (CYAs) aged 2–15 years.

**Material and methods:**

This was a questionnaire‐based, interview‐style community survey and on‐site practical assessment of CYAs' toothbrushing skills conducted during two 1‐day public science‐promoting events held at a major German university hospital in consecutive years. CYAs first answered questions on toothbrushing frequency, dental aids used, and dental care. They subsequently underwent diagnostic staining and demonstrated their brushing technique and method. CYAs' responses (percentages) to questionnaire items addressing oral hygiene knowledge and practice, and on‐site assessment of toothbrushing skills served as the main outcome measures.

**Results:**

Of 244 participating CYAs, 178 (73%) CYAs had no caries experience, the percentage increasing with age from 5% in 2–5‐year‐olds to 40% in those aged > 10 years. Of 117/244 (48%) indicating that teeth should be brushed three times daily, 80/117 (68%) self‐reported twice‐daily brushing, 32/117 (27%) reported brushing three times, and 4/117 (3%) stated doing so only once. Although 131/244 (54%) considered that teeth should be brushed for 3 min, 77/131 (59%) self‐reported actually doing so and 41/131 (31%) reported brushing for 2min. Seventeen of 42 (40%) participants aged > 10 years showed no systematic brushing method, with 21/42 (50%) failing to clean their teeth completely. Participants aged 6–10 years exhibited the highest proportion (97/134, 72%) of complete cleaning. One hundred and forty‐six of 244 (60%) of CYAs knew about floss; 63/134 (43%) reported using it. Good adherence to oral health recommendations (i.e., brushing ≥ 2/day for ≥2min) was observed in 212/244 (87%) CYAs, the remaining 32/244 (13%) exhibiting poor adherence.

**Conclusion:**

CYAs knew about the importance of oral hygiene and cleaned their teeth frequently. However, translation of their knowledge into practice showed deficiencies. Repeated encouragement to put oral health knowledge into practice is important.

## 1 INTRODUCTION

Daily oral hygiene is a major challenge that begins in early childhood and some children resist brushing their teeth or find it annoying at times (Makuch et al., 2011). In Germany, statutory health insurance offers dental screening examinations from age 6 months (National Association of Statutory Health Insurance Dentists [KZBV], National Association of Statutory Health Insurance Funds [GKV‐Spitzenverband], 2019). However, dental caries in children is among the 10 highest‐incidence diseases worldwide (GBD 2016 Disease and Injury Incidence and Prevalence Collaborators, 2017). Sugary foods cause microorganisms in the plaque biofilm to form organic acids, which by demineralizing the tooth enamel contribute to caries formation (van Waes & Stöckli, 2001). Moreover, gingival diseases, particularly gingivitis, also develop in the presence of biofilms and are as frequent in children as in adults (Kasaj & Willershausen, 2013). Clinically, gingivitis causes swelling, redness, and tenderness. Diagnostically, bleeding is evident on probing (Caton et al., 2018). Initial caries can be arrested (Young et al., 2015) and gingival inflammation can be prevented by oral hygiene (Birch et al., 2015). Appropriate prophylactic measures include fluoridation, oral hygiene instruction, dietary guidance, and fissure sealing (Birch et al., 2015; Toumba et al., 2019). To ensure dental prophylaxis is available to children nationwide, preschool and school prevention programs have been implemented in Germany. Furthermore, individual prophylactic measures are offered as part of dental examinations, based on Book V of the German Social Code (Sozialgesetzbuch Fünftes Buch [SGB V]). With dental prophylaxis and care available to the whole population, Germany has seen marked decreases in caries prevalence over the years (Jordan & Micheelis, 2016). However, although their caries experience has decreased, children and adolescents still have the highest prevalence of carious lesions (Schenk & Knopf, 2007), with caries being more frequent in socially disadvantaged children and children with migrant backgrounds (Brunner‐Strepp, 2001; Jordan & Micheelis, 2016; Kühnisch et al., 2003; Rajab & Hamdan, 2002; Sundby & Petersen, 2003; Taani, 2002). Against this backdrop, the present interview‐style, questionnaire‐based study aimed to investigate knowledge about oral hygiene, relevant self‐reported behavior, and practical toothbrushing ability in children and young adolescents (CYAs) in the context of caries prevalence being highest in the general pediatric population.

## 2 METHODS

### 2.1 Study design

This was a community‐based, two‐part pediatric oral hygiene study encompassing (1) completion of a questionnaire and (2) a practical assessment of toothbrushing behavior. The questionnaire was designed to record the participants' knowledge of the subject and their self‐reported behavior, whereas the subsequent practical part served to assess the extent of the actual toothbrushing behavior. Participants' age and gender were recorded but no participant identifiable data were collected. Participants were interviewed once only.

### 2.2 Participants and setting

CYAs aged 2–15 years attending the “Long Night of Science” event at Saarland University Medical Centre in 2016 and 2017 were invited spontaneously to participate on site. The only exclusion criterion was refusal of parental consent.

### 2.3 Interventions

Spontaneous on‐site, interview‐style completion of a questionnaire with parental assistance if required, followed by an assessment of toothbrushing behavior in terms of participants' brushing technique and systematic method of brushing as detailed below.

### 2.4 Oral health and hygiene knowledge

Answers were not predefined in the questionnaire documentation sheet. Rather, children responded freely, with answer categories being assigned by the interviewer in the process. This was important to ensure that subsequent analysis could establish whether oral hygiene knowledge and self‐reported toothbrushing behavior correlated. The first set of questions addressed reasons for toothbrushing and the potential consequences of neglecting it. Participants were also asked, inter alia, how often and for how long teeth needed to be brushed.

### 2.5 Self‐reported behavior

The second phase recorded the self‐reported frequency and duration of toothbrushing, use of dental floss, frequency of dental check‐ups, and other behavioral items on the questionnaire (latter data not shown). Parents of 2–5‐year‐olds were asked to assist their children in answering questions. If questions were not answered due to young age, this was recorded.

### 2.6 Technique and systematic method of toothbrushing

After finishing the interview part, children were asked to brush their teeth on site. Toothbrushing thoroughness was assessed in terms of dental plaque removal after staining with a prebrushing dye (Mira‐2‐Ton two‐tone plaque revealer, Hager & Werken) before assessing children's technique and systematic method of toothbrushing. Children were observed while brushing and it was recorded whether they brushed all surfaces completely or incompletely, and whether they did so systematically, for example, clockwise. Brushing technique was recorded as, for example, “scrubbing” or circular brushing, and it was recorded whether toothbrushing was performed methodically, that is, in a specific sequence, for instance starting with the occlusal surface, followed by the front surfaces and finally the rear surfaces of the teeth, a method known in Germany as the “Kauflächen, Außenflächen, Innenflächen (KAI; occlusal surfaces, external surfaces, internal surfaces) method”. Children were not given any brushing instructions in advance so as not to interfere with their spontaneous toothbrushing behavior. Subsequently, children received toothbrushing training and were encouraged to brush their teeth according to standard recommendations.

### 2.7 Data analysis

Data analysis utilized IBM SPSS 24 (IBM Deutschland GmbH). Responses to questions were evaluated both pooled for all ages and/or by age groups of 2–5, 6–10, and >10 years. Comparative questions were analyzed with regard to toothbrushing knowledge and toothbrushing behavior with and without categorization by age. To analyze children's toothbrushing technique, they were divided into two groups based on oral hygiene behavior: (1) those showing good adherence to oral hygiene recommendations, defined as complete congruence between knowledge and self‐reported behavior for brushing frequency ≥ 2/day and brushing duration ≥ 2 min; and (2) all others were categorized as showing poor adherence.

The *χ* ^2^ test was used to statistically analyze the relationship between toothbrushing technique and age; the comparison of knowledge with self‐reported behavior; and the relationship between knowledge, behavior and toothbrushing technique, and toothbrushing adherence. Values of *p* < .05 were considered statistically significant.

### 2.8 Ethics approval

Ethics approval for this study was obtained from the Ethics Committee of the Saarland Medical Association (Approval number 188/19).

## 3 RESULTS

### 3.1 Participant characteristics

All 244 voluntary participants completed the interview‐style questionnaire and practical toothbrushing assessment. Caries experience by age group (2–5, 6–10, and >10 years) is shown in Table 1. Caries increased with age, with 20/50 (40%) children aged > 10 years reporting experience of caries.

**Table 1:** Participant characteristics by age group

	Age groups			
	2–5 years	6–10 years	>10 years	All
Participants	38 (16%)	156 (64%)	50 (20%)	244 (100%)
No caries experience	36/38 (95%)	112/156 (72%)	30/50 (60%)	178/244 (73%)
Caries experience	2/38 (5%)	44/156 (28%)	20/50 (40%)	66/244 (27%)

### 3.2 Oral health knowledge

**Figure 1:**
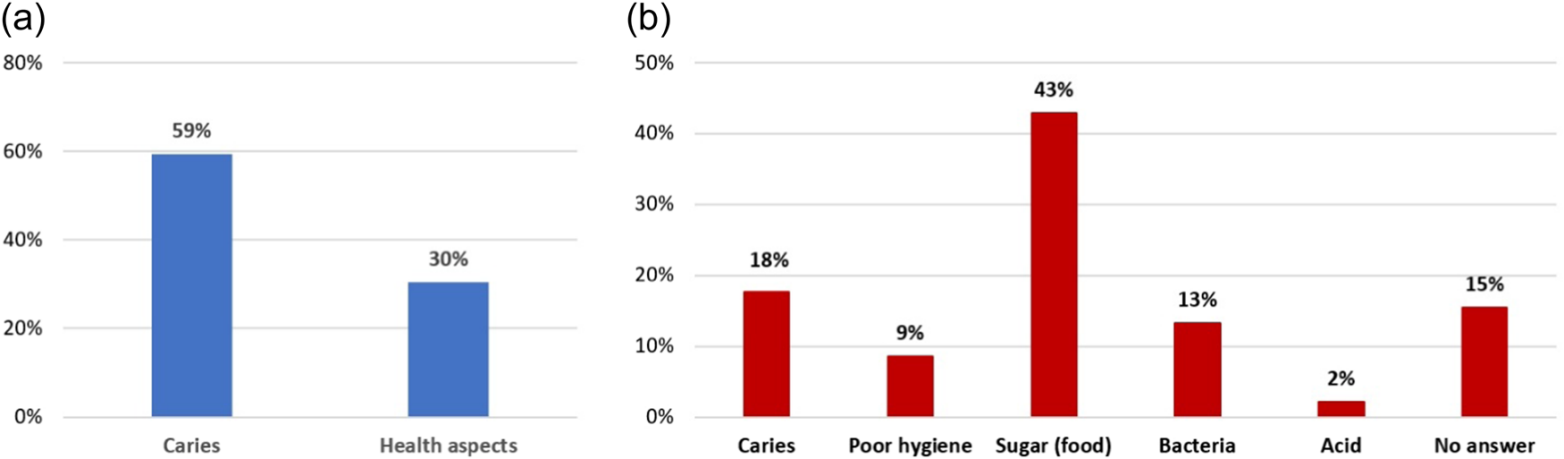
General oral hygiene and health questions. (a) Why should you brush your teeth? (b) Do you know what makes teeth sick?

When asked “why should you brush your teeth,” 145/244 (59%) children gave “caries” as the reason (Figure 1a). Asked “what makes teeth sick,” 100/233 (43%) children answered “sugar” and 41/233 (18%) answered “caries,” whereas lack of hygiene and bacteria combined were mentioned by 51/233 (22%) children (Figure 1b).

### 3.3 Knowledge versus self‐reported behavior

**Figure 2:**
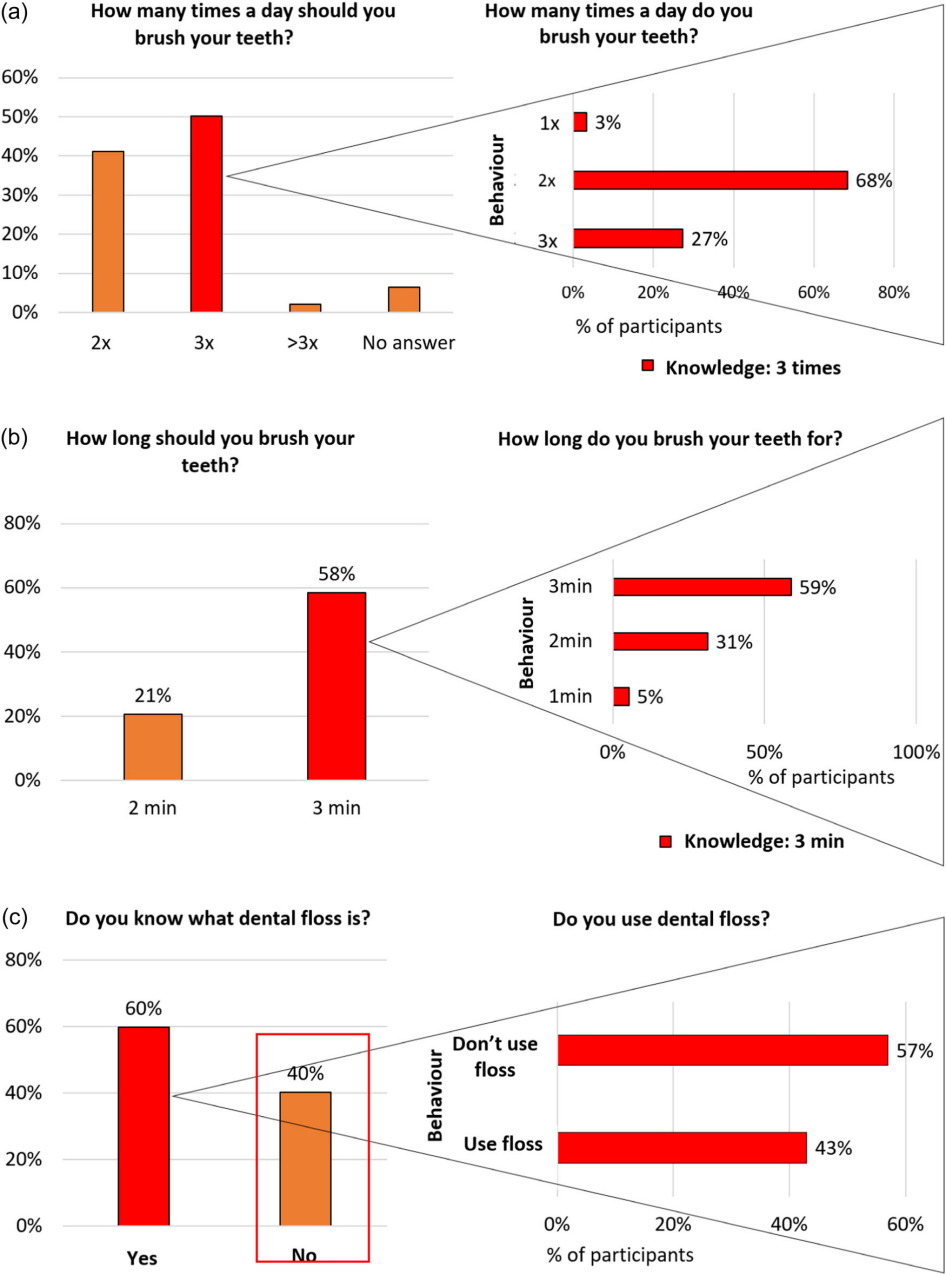
Knowledge versus self‐reported behavior. (a) Daily frequency of toothbrushing.  (b) Duration of toothbrushing. (c) Dental floss.

Almost half (117/244, 48%) of all children indicated that teeth should be brushed three times daily; 80/117 (68%) self‐reported twice‐daily brushing, 32/117 (27%) indicated they cleaned their teeth three times, and 4/117 (3%) stated they did so only once daily. The difference between knowledge and self‐reported behavior was statistically significant (*p* = .002; Figure 2a).

Of all 244 children, 131 (54%) answered that teeth should be brushed for 3 min, with 77/131 (59%) indicating they actually brushed their teeth that long. Forty‐one of 131 (31%) and 7/131 children (5%) self‐reported brushing for 2 and 1 min, respectively. The difference between knowledge and reported behavior was statistically significant (*p* < .001; Figure 2b).

Of all children, 146 (60%) knew about flossing, 63 (43%) of whom self‐reported using it, a statistically significant difference (*p* < .001; Figure 2c).

### 3.4 Toothbrushing technique and method by age

Across age groups, a toothbrushing technique and systematic cleaning was noted in 113/213 (53%), with 133/213 (62%) children achieving complete cleaning.

**Figure 3:**
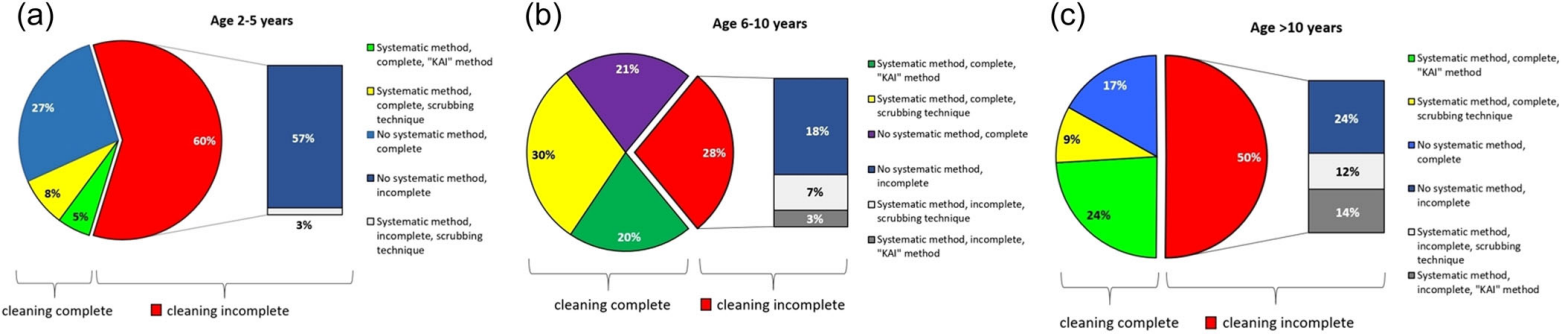
Technique and systematic method of toothbrushing by age group. (a) 2–5 Years. (b) 6–10 Years. (c) >10 Years.

Figure 3 shows by age group the proportions of children exhibiting complete versus incomplete brushing of their teeth during the toothbrushing assessment, and further breaks down the two main categories according to technique and systematic method observed.

In 2–5‐year‐old children, toothbrushing was complete in 15/37 (40%) and incomplete in 22/37 (60%) of children, with 31/37 (84%) showing no discernible systematic method of brushing. Among 6–10‐year‐olds, brushing was complete in 97/134 (72%) and incomplete in 37/134 (28%), with no discernible systematic brushing method in 52/134 (39%) children. In children aged > 10 years, complete brushing and incomplete brushing were both 21/42 (50%), with 17/42 (40%) children exhibiting no systematic toothbrushing method.

As regards incomplete brushing, 22/37 (60%) 2–5‐year‐old children brushed their teeth incompletely, compared with 37/134 (28%) 6–10‐year‐olds (*p* = .001) and 21/42 (50%) >10‐year‐olds (*p* = .498). Thus, the difference between the youngest and the intermediate age group was statistically significant, whereas the difference between the youngest and the oldest age group was not.

However, with regard to completeness of brushing, the proportion of 72% of 6–10‐year‐old children was significantly higher than the 50% of those aged > 10 years (*p* = .014). Thus, compared with the oldest age group, more intermediate‐age children cleaned their tooth surfaces completely.

### 3.5 Adherence groups

Table 2 summarizes the comparisons for the good adherence group of 212/244 (87%) and the poor adherence group of 32/244 (13%) participants regarding knowledge and self‐reported toothbrushing behavior. Good adherence was associated with statistically significant differences between knowledge and self‐reported behavior regarding dental floss awareness (68% vs. 29%) and having ≥2 dental check‐ups yearly (75% vs. 63%). The poor adherence group showed no statistically significant differences between knowledge and self‐reported behavior for brushing frequency ≥ 2/day and brushing duration ≥ 2 min but did exhibit significant differences for dental floss awareness (25% vs. 13%) and ≥2 dental check‐ups yearly (47% vs. 31%).

**Table 2:** Comparison of adherence groups in respect of knowledge and self‐reported toothbrushing behavior

	Good adherence (*n* = 212)			Poor adherence (*n* = 32)		
Questionnaire	Knowledge	Self‐reported behavior	*p*	Knowledge	Self‐reported behavior	*p*	
Brushing frequency ≥ 2/day	195/212 (92%)	195/212 (92%)	–	28/32 (88%)	24/32 (75%)	0.422	
Brushing duration ≥ 2 min	186/212 (88%)	186/212 (88%)	–	20/32 (63%)	4/32 (13%)	0.776	
Awareness of dental floss	145/212 (68%)	62/212 (29%)	**0.001**	8/32 (25%)	4/32 (13%)	**0.005**	
Dental check‐ups ≥ 2/year	159/212 (75%)	134/212 (63%)	**0.001**	15/32 (47%)	10/32 (31%)	**0.026**	

*Note*: Knowledge versus self‐reported behavior in children with good (*n* = 212) and poor (*n* = 32) adherence to recommended oral hygiene practices. Percentages are based on the size of the respective adherence group. Bold *p* values indicate statistical significance.

As regards actual toothbrushing behavior on‐site, brushing was incomplete in 70/212 (33%) and 12/32 (38%) participants in the good and the poor adherence group, respectively. Similarly, 84/212 (40%) and 18/32 (56%) in the respective adherence groups exhibited no systematic cleaning method.

## 4 DISCUSSION

This community‐based study in CYAs aged 2–15 years collected empirical data using an interview‐style, purposely redundant questionnaire covering oral health and hygiene knowledge topics and self‐reported behavior, followed by an on‐site practical assessment of the participants' actual toothbrushing technique and method. We also analyzed adherence to oral health and hygiene recommendations.

Caries experience is not evenly distributed among children in Germany, with 9% of children accounting for 60% of early carious teeth (Jordan & Micheelis, 2016), as confirmed by other pediatric oral health studies (Jordan & Micheelis, 2016; Pieper & Schulte, 2004; Reinhardt et al., 2010). The majority (73%) of our participants had no experience of caries‐related invasive dental treatment. This finding reflects epidemiological data from Germany reported in 2016, according to which 81% of 12‐year‐old children were free of caries (Jordan & Micheelis, 2016), compared with 70% in its 2006 predecessor (Micheelis & Schiffner, 2006).

Only 3% of our study participants self‐reported brushing their teeth less often than twice daily, in contrast to 26% of 3–17‐year‐olds, as reported by the large KiGGS survey (Schenk & Knopf, 2007). This may be due to the “Long Night of Science” event generally being attended predominantly by children and health‐conscious parents.

Regarding toothbrushing frequency, whilst half of our participants considered teeth should be brushed three times daily, only 27% self‐reported doing so; 68% reported brushing twice and 3% only once daily. This finding points to a discrepancy between knowledge and self‐reported behavior but also shows that brushing teeth several times daily was firmly established in our cohort. To our knowledge, no other study has directly compared with oral health knowledge and actual toothbrushing behavior in children.

As regards toothbrushing duration, 59% of participants reported brushing their teeth for 3 min. By contrast, a study in 114 5–13‐year‐old children found that only 29% brushed for ≥3 min (Skaisgirski, 2010). Our study design was unable to directly distinguish whether participants were very health conscious or unable to correctly estimate brushing time. To assess the reliability of participants' statements, we compared their knowledge and self‐reported behavior. About half of children's answers were considered reliable based on 54% of children, indicating that teeth should be brushed for 3 min, of whom just under 60% self‐reported brushing for 3 min, another 31% self‐reporting brushing time as 2 min, and 5% as 1 min.

Across all age groups, 60% of participants knew about dental floss, but only 43% self‐reported using floss, thus revealing a discrepancy between knowledge and self‐reported behavior. By comparison, a 1998 questionnaire‐based study investigating the flossing habits of 41,142 12–16‐year‐old secondary school pupils in England found that just 8% of respondents reported flossing every day, another 29% reporting occasional use (Macgregor et al., 1998).

Our on‐site assessment of toothbrushing behavior found that 60% of 2–5‐year‐olds cleaned the tooth surfaces incompletely and 84% showed no systematic brushing method. These findings may reflect incompletely developed wrist mobility in children around the age of 3 years, limiting them to large back and forth movements at this stage. Later, circular movements from the elbow become possible, making cleaning easier.

Of the 6–10‐year‐olds assessed on site, 72% brushed their teeth completely and 39% exhibited no systematic method. Improved brushing at this age is plausibly attributable to an increased ability to perform fine motor movements. Comparable literature results for this age group are lacking. However, caries experience in our cohort increased from 5% in 2–5‐year‐olds to 28% in 6–10‐year‐olds. Possibly, this increase might be due to altered dietary behavior or the fact that the latter age group was the largest in our cohort.

Of participants aged > 10 years, 62% cleaned their teeth completely and 53% brushed systematically. Participants in this age group also brushed their teeth less systematically than did the 6–10‐year‐olds. The poorer performance observed in >10‐year‐olds relative to 6–10‐year‐olds may be explained by different rates of parental checking, which were 28% and just over 50%, respectively.

Furthermore, caries experience increased with age from 5% in 2–5‐year‐olds to 40% in >10‐year‐olds in our study, that is, almost half of >10‐year‐olds had caries experience. Poorer toothbrushing behavior in >10‐year‐olds thus reflects in their caries experience. At this age, adolescents generally seek to gain more independence, resulting in toothbrushing being checked less frequently by parents. Consumption behavior also changes as children approach adolescence (Taani, 2002).

The Fifth German Oral Health Study found the pattern of toothbrushing to be inadequate in the majority of children based on a composite behavioral index incorporating daily frequency, brushing ≥2 times daily, timing and brushing for ≥2 min (Jordan & Micheelis, 2016). Inadequate toothbrushing was reported for 55% of 12‐year‐olds compared with 58% and 73% in the Fourth and the Third German Oral Health Study, respectively (Micheelis & Schiffner 1999, 2006). Overall, this trend suggests that children's toothbrushing behavior in Germany has improved over the years (Jordan & Micheelis, 2016), a finding consistent with our results (Jordan & Micheelis, 2016).

Lastly, analysis of adherence to recommendations on complete and systematic brushing revealed that compared to knowledge about flossing and twice‐yearly dental check‐ups, the frequencies of corresponding behaviors were significantly lower in the poor adherence group, whereas in the good‐adherence group this was noted only for flossing.

Advantage of our study design and research approach include the voluntary nature of participation. Children were motivated and positive in their attitude. However, this also potentially involved a positive selection bias. Thus, our cohort may not have been a representative sample of the corresponding general‐population age group. Children aged 6–10 years accounted for the largest proportion of our pediatric cohort. This may indicate that this age group had the greatest interest in the “Long Night of Science” oral health event, and that oral hygiene education can be communicated in a playful way. Lastly, children aged 2–5 years had difficulty fully answering the questions despite parental assistance and therefore may have been influenced by their parents.

## 5 CONCLUSIONS

Our study shows that children attach great importance to oral hygiene and clean their teeth regularly, with 6–10‐year‐olds brushing best. Nonetheless, there are shortcomings in their ability to translate knowledge into practice. Children should therefore regularly be encouraged to utilize their oral hygiene knowledge and adhere to general oral hygiene recommendations.

## AUTHOR CONTRIBUTIONS

Madline P. Gund, Marina Bucher, Matthias Hannig, Stefan Rupf, and Tilman R. Rohrer conceived the study and designed the questionnaire. Marina Bucher and Madline P. Gund conducted the survey. All authors contributed to data analysis and interpretation. Madline P. Gund, Tilman R. Rohrer, and Stefan Rupf prepared the draft manuscript. All authors contributed to and reviewed the manuscript. All authors read and approved the final manuscript. An English translation of the study questionnaire is available online.

## CONFLICT OF INTEREST

The authors declare no conflict of interest.

## ETHICS STATEMENT

All procedures performed in studies involving human participants were in accordance with the ethical standards of the institutional and national research committee, and with the 1964 Helsinki declaration and its later amendments or comparable ethical standards. Ethics approval for this study was obtained from the Ethics Committee of the Saarland Medical Association (Approval number 188/19). Written informed consent was not required and not obtained as study interventions were conducted anonymously and no person‐identifiable data were collected. All questionnaire‐based interviews and practical assessments were carried out in the presence of the participants' parents or guardians.

## Data Availability

The data that support the findings of this study are available from the corresponding author upon reasonable request.
